# Waist circumference shows a significant association with the risk of noncommunicable diseases: a cross-sectional study from a large community sample in Southeast China

**DOI:** 10.1186/s12889-026-28417-7

**Published:** 2026-07-09

**Authors:** Qi Lin, Wenting Tang, Ze Yang, Xin Fang, Lei Li, Jianjun Xiang, Xiangju Hu

**Affiliations:** 1 Fujian Health College, Fuzhou, 350101 Fujian China; 2 The Affiliated Zhangzhou Center for Disease Control and Prevention of Fujian Medical University, Zhangzhou, Fujian 363000 China; 3 Department for Chronic and Noncommunicable Disease Control and Prevention, Fujian Provincial Center for Disease Control and Prevention, Fuzhou, Fujian 350012 China; 4 School of Public Health, Xiamen University, Xiamen, Fujian 361102 China; 5 Department of Preventive Medicine, School of Public Health, Key Laboratory of Environment and Health, Fujian Medical University, Fuzhou, Fujian 350122 China

**Keywords:** Waist circumference, Noncommunicable diseases, Central obesity, Multimorbidity

## Abstract

**Objective:**

To study the magnitude and pattern of the cross-sectional associations of body mass index (BMI) and waist circumference (WC) with the prevalence of hypertension (HTN), type 2 diabetes (T2DM), and dyslipidemia in a large community-dwelling population in Southeast China.

**Methods:**

A total of 135,352 permanent residents aged 35–75 years were recruited using multistage stratified cluster random sampling from Fujian Province, China (2018–2021). Physical measurements included height, weight, WC, and blood pressure. Fasting fingertip blood samples were tested for plasma glucose and lipid profiles. Overweight/obesity was defined as BMI ≥ 24 kg/m², and central obesity was defined as WC ≥ 85 cm for males and ≥ 80 cm for females. Multiple linear regression with Z-score standardization was used to directly compare standardized effect sizes of BMI and WC on continuous metabolic parameters. Modified Poisson regression with robust error variance was applied to estimate adjusted risk ratios (aRR) for HTN, T2DM, dyslipidemia, and multimorbidity burden, adjusting for sociodemographic and lifestyle factors.

**Results:**

Among 135,352 participants (mean age 56.7 ± 9.9 years; 59.5% female), the prevalence of HTN, T2DM, and dyslipidemia was 46.2%, 18.3%, and 16.6%, respectively. After Z-score standardization, every 1-SD increase in WC was associated with a larger increase in systolic blood pressure (β = 0.132 vs. β = 0.115 for BMI) and far larger elevations in fasting plasma glucose (β = 0.130 vs. β = 0.020) and triglycerides (β = 0.176 vs. β = 0.091), as well as a stronger inverse association with HDL-C (β=–0.185 vs. β=–0.147). All obesity subtypes independently increased risks of HTN (mixed obesity aRR = 1.926, isolated elevated BMI aRR = 1.434, isolated elevated WC aRR = 1.358), T2DM (mixed obesity aRR = 1.574, isolated elevated WC aRR = 1.495, isolated elevated BMI aRR = 1.143), and dyslipidemia (mixed obesity aRR = 1.402, isolated elevated BMI aRR = 1.304, isolated elevated WC aRR = 1.102). Elevated WC alone (aRR = 1.432), elevated BMI alone (aRR = 1.409), and mixed obesity (aRR = 1.973) independently raised the multimorbidity burden. Age was the strongest independent correlate of all outcomes. Smoking showed a spurious “protective” association, likely due to healthy survivor bias and residual confounding.

**Conclusions:**

Both BMI and WC are independently associated with HTN, T2DM, and dyslipidemia, with WC notably associated with T2DM. Mixed obesity poses a high risk and nearly doubles multimorbidity burden. Integrating WC into routine screening alongside BMI is recommended to identify central obesity risks. Targeted interventions should focus on high-risk groups. Future studies are needed to establish causality and evaluate WC-centered programs for reducing the NCD burden.

**Supplementary Information:**

The online version contains supplementary material available at https://doi.org/10.1186/s12889-026-28417-7.

## Introduction

Globally, there has been a significant increase in disability-adjusted life years (DALYs) attributable to metabolic risk factors and non-communicable diseases due to population growth and aging [1]. Ischemic heart disease and stroke remain the leading causes of death, the probability of mortality before a certain age has declined across regions [2]. As the world’s most populous middle-income country, China is undergoing rapid aging and urbanization. In 2023, NCDs were the leading contributor to the disease burden among all Level 1 causes in China [3]. The weighted prevalence of hypertension (HTN) reached 31.6% in 2021–2022, an 8.4% absolute increase from 2012 to 2015 [4]. The overall prevalence of dyslipidemia among teachers in China was 38% [5]. During the past 40 years, the prevalence of diabetes in China has increased from less than 1.0% in 1980 to 12.4% in 2018 [6]. 

Currently, there are more studies on the relationship between obesity and NCDs globally. Notably, studies have found that over 70% of NCDs have a documented association with obesity [7]. Obesity is typically classified into central obesity and general obesity. Body mass index (BMI) primarily reflects general obesity, while waist circumference (WC) specifically captures visceral adipose tissue. Most research is limited to the use of BMI alone. But visceral white adipose tissue (vWAT) exhibits a stronger association with metabolic diseases, particularly insulin resistance (IR), chronic inflammation, metabolic syndrome, and cardiovascular diseases [8]. A larger WC was strongly and linearly associated with the risk of T2DM, HTN, elevated blood pressure, various cardiovascular diseases, and all-cause mortality [9–11]. Therefore, we propose the hypothesis that WC exhibits significant associations with HTN, T2DM, and dyslipidemia in this Southeast Chinese population. Furthermore, we explored whether central obesity confers excess risk even among individuals with a normal BMI.

## Participants and methods

### Participants

**Fig. 1:**
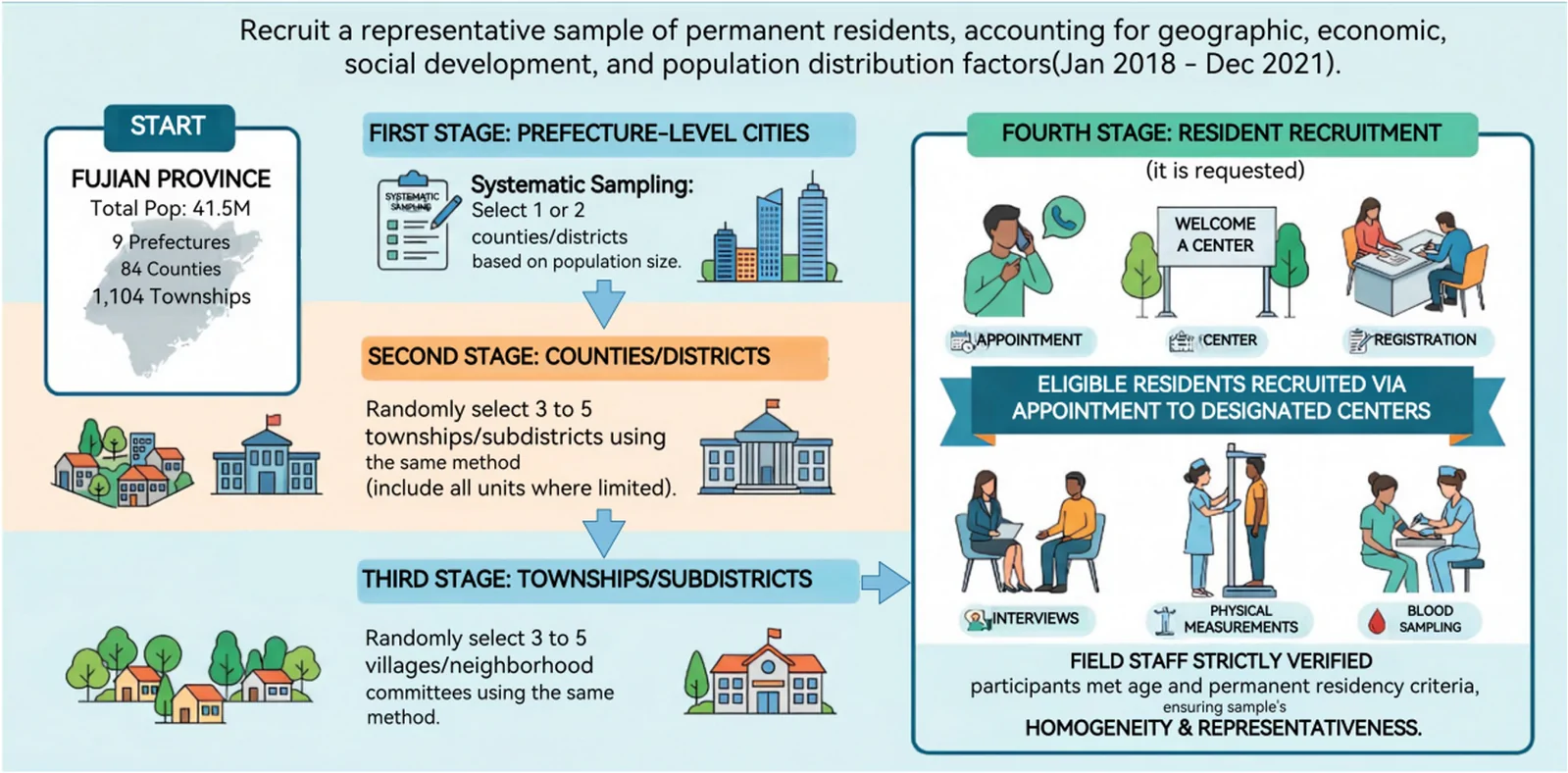
Multistage stratified cluster random sampling in Fujian Province, China

We used multistage stratified cluster random sampling to recruit a representative sample of permanent residents, accounting for geographic, economic, social development, and population distribution factors. Fujian Province has a total population of approximately 41.5 million (2020 census) and comprises 9 prefecture-level cities, 84 counties/districts, and 1,104 townships/subdistricts. The survey was conducted from January 2018 to December 2021, with a four-stage sampling procedure: First, one or two counties/districts were selected from Fujian Province’s prefecture-level cities using systematic sampling based on population size. Second, 3 to 5 townships or subdistricts were randomly selected within these units using the same method, including all units where numbers were limited. Third, 3 to 5 villages or neighborhood committees were similarly selected within the chosen townships or subdistricts. Finally, eligible residents were recruited via appointment to designated centers for registration, interviews, physical measurements, and blood sampling. Field staff strictly verified that all participants met the age and permanent residency criteria, ensuring the sample’s homogeneity and representativeness (Fig. 1).

The incidence of three chronic diseases—dyslipidemia, HTN, and T2DM—follows a clear age-related increasing pattern: the average diagnosis ages for these three conditions are 55.1 years, 55.4 years, and 56.7 years, respectively, all concentrated in the middle and elderly age groups [12]. Moreover, the risk of developing these diseases rises rapidly with advancing age. Therefore, the eligibility criteria included age between 35 and 75 years, permanent residency (living in the country for more than six months), which would ensure effective coverage of the population most burdened by the disease, thereby maximizing benefits.

The study initially enrolled 135,400 participants, of whom 135,352 were ultimately included after screening. The enrolled participants ranged in age from 35 to 75 years. A total of 48 individuals were excluded due to incomplete information or age mismatch. The participation rate was 99.96%. Participants were selected for in-person questionnaires, physical examinations, and laboratory tests. The participants were required to present their identification at community plazas or health-care centers for verification. Written informed consent was obtained from all participants at the onset of the project. Following residency verification, those who had signed the informed consent agreement were enrolled.

### Methods

We standardized all protocols, questionnaires, and training procedures. Additionally, we strictly calibrated all measurement tools, including sphygmomanometers, tape measures, and laboratory meters.

Trained and qualified investigators collected demographic information (gender, age, nationality, education, family annual income, marital status, and medical insurance) and lifestyle data (smoking and drinking) from participants via face-to-face questionnaires [13].

After the survey, 5% of the respondents at each monitoring site were randomly selected for key information review, resulting in a 99% consistency rate with the initial responses.

The physical measurements included height, weight, WC, and blood pressure. Height and weight were assessed via a meter (GMCS-I, Jianqiao Medical Equipment Co., Ltd., China) with a 2.0 m range and 0.1 cm accuracy and a scale (RGT-140; manufacturer: Shanghai Rongtao Medical Equipment Co., Ltd., China) with a 150 kg range and 0.1 kg accuracy. WC was measured using a tape with a 1.5 m range and 0.1 cm accuracy. BMI was calculated via the following formula: BMI = weight (kg)/height (m^2^). Blood pressure was measured after the participants had rested for 5 min, and two readings were taken. If the SBP difference exceeded 10 mm Hg (1 mm Hg = 0.133 kPa), a third measurement was taken, and the average of the last two readings was recorded. An electronic sphygmomanometer (HEM-7430, Omron Corporation, Japan) was utilized for blood pressure assessments. According to the definition of hypertension, measurements must be taken on three separate days.

The laboratory tests included fasting plasma glucose (FPG) and lipid profiles. Fasting fingertip blood samples were collected by nurses from local district hospitals or community health service centers. FPG was measured via an electrochemical biosensor with a blood glucose meter (Baijie PD-G001, Qinli Company, China), whereas total cholesterol (TC), low-density lipoprotein cholesterol (LDL-C), high-density lipoprotein cholesterol (HDL-C), and triglycerides (TG) were assessed via the CardioCheck PA Rapid Lipid Test (1700; PTS Diagnostics, Indianapolis, IN, USA). The Baijie PD-G001 glucose meter (electrochemical biosensor) has been validated against the venous hexokinase method in Chinese populations, with a reported correlation coefficient *r* > 0.95 and a mean absolute relative difference < 5% (validation reference added). The CardioChek PA lipid analyzer (model 1700) has shown good agreement with standard enzymatic methods for TC, TG, HDL-C, and LDL-C in multi-center validation studies, with inter-device coefficients of variation ranging from 3% to 8% depending on the lipid fraction. Daily quality control checks were performed using manufacturer-provided control solutions for both devices before each day’s measurements.

Blood sample collection procedure:

- Prepare and verify the functionality of all testing instruments.
- Seat participants, disinfect the lateral side of the left fingertip with 75% alcohol, and allow it to air dry.
- Activate the blood glucose meter and insert a test strip. Puncture the fingertip with a sterile lancet, express a drop of blood by squeezing from the palm root to the fingertip, and apply the blood to the test strip. Press the puncture site with a cotton swab until bleeding stops.
- Record and register results.

The central ethics committees at the China National Center for Cardiovascular Disease (NCCD) and the Fuwai Hospital approved this project (Ethics Approval Number: 2014–574).

### Criteria of diagnosis and definitions

Overweight/Obesity: BMI ≥ 24 kg/m² [14].

Central obesity: WC ≥ 85 cm for males, WC ≥ 80 cm for females [14]. 

HTN: SBP ≥ 140 mmHg and/or DBP ≥ 90 mmHg on more than three separate occasions on a non-same day, or a clear diagnostic record from hospitals grade two or above [15]. 

T2DM: FPG ≥ 7.0 mmol/L or a clear diagnostic record from hospitals grade two or above.

Dyslipidemia: the presence of any one of the following: TC ≥ 6.22 mmol/L, LDL-C ≥ 4.14 mmol/L, HDL-C < 1.04 mmol/L, TG ≥ 2.26 mmol/L, or a clear diagnostic record from hospitals grade two or above [16]. 

Multimorbidity: the coexistence of two or more chronic conditions in the same individual, without designating any one as the index disease [17].

In this study, for blood pressure measurement in the field, only a single visit was conducted. During that single visit, we performed two parallel measurements (if the difference exceeded 10 mmHg, a third measurement was taken, and the average of the last two readings was recorded). This procedure does not constitute the “three separate occasions on non-same days” required for clinical diagnosis; rather, it was used to describe the current blood pressure level and to estimate prevalence. The actual determination of hypertension prevalence combined two sources: the single-visit field blood pressure measurement (reflecting current status) and a clear diagnostic record.

We did not routinely distinguish T2DM from other forms of diabetes (e.g., type 1 diabetes, gestational diabetes, or secondary diabetes), because the study population consisted of adults aged 35–75 years, in whom T2DM accounts for more than 95% of all diabetes cases in China [14]. Moreover, the national surveillance protocol does not require antibody or C-peptide testing. Therefore, the term ‘T2DM’ in this manuscript refers to diagnosed diabetes in this age group, which is overwhelmingly T2DM.

Disease definitions combined objective measurements and self-reported diagnoses. The Kappa coefficients were 0.355 for hypertension and 0.481 for T2DM, indicating moderate agreement; for dyslipidemia, Kappa was only 0.046 (very low agreement), but its diagnosis relied predominantly on objective measurements (36.4%), thus limiting potential bias. Based on these distributions, we further performed sensitivity analyses. Sensitivity analysis demonstrated that the outcome misclassification bias had minimal impact on the primary conclusions.

### Statistical analysis

For practical and quality control, data were double-entered using EpiData 3.1 (EpiData Association, Odense, Denmark) with range and consistency checks. Data cleaning procedures included checking for duplicate records, verifying age eligibility (35–75 years), and logical consistency checks (e.g., SBP > DBP, plausible ranges for height, weight, WC, FPG, and lipid parameters), and identifying extreme values (beyond ± 4 SD). A 5% random sample of questionnaires was reviewed for consistency, achieving 99% agreement. After cleaning, 48 individuals were excluded due to incomplete information or age mismatch, resulting in a final analytic sample of 135,352 participants. After data cleaning, all statistical analyses (descriptive statistics, linear regression, modified Poisson regression, etc.) were performed using SPSS 26.0 (IBM Corp., Armonk, NY, USA). Given the large sample size $\left(\mathrm{n}=135,352\right)$, parametric methods were applied based on the central limit theorem.

Multiple linear regression analyses were conducted to identify factors independently associated with BMI and WC (as continuous dependent variables). All covariates were selected based on prior literature and clinical knowledge; no data-driven variable selection methods were applied. Collinearity diagnostics were assessed using the variance inflation factor (VIF), with VIF < 5 indicating no serious multicollinearity. Residual independence was evaluated using the Durbin-Watson test, with values between 1.5 and 2.5 considered acceptable. Model fit was evaluated using adjusted R². Results are reported as unstandardized regression coefficients (B) with 95% confidence intervals (CI), and standardized coefficients (β).

To directly compare the relative strength of associations between the two obesity indicators (BMI and WC) and continuous metabolic parameters $\left(\mathrm{SBP},\;\mathrm{DBP},\;\mathrm{FPG},\;\mathrm{TC},\;\mathrm{TG},\;\mathrm{HDL-C},\;\mathrm{and}\;\mathrm{LDL-C}\right)$, separate multiple linear regression models were constructed for each metabolic parameter as the dependent variable. BMI and WC were entered as independent variables after Z-score standardization, allowing the standardized coefficients (β) to represent the change in the outcome per 1-SD increase in each obesity indicator. This approach enabled direct comparison of the effect sizes of BMI and WC within the same model.

Given the high prevalence of NCDs, modified Poisson regression with robust error variance (GENLIN procedure) was employed to estimate adjusted risk ratios (aRR) and 95% CI for the associations between obesity phenotypes and each NCD. Obesity was classified into four phenotypes based on BMI and WC cutoffs: (1) both normal (reference group), (2) isolated WC elevation (normal BMI but elevated WC), (3) isolated BMI elevation (elevated BMI but normal WC), and (4) mixed obesity (both elevated). Given the significant positive correlations among the three diseases (all pairwise *p* < 0.001), each disease model additionally adjusted for the presence of the other two conditions to obtain more robust independent effect estimates.

For the multimorbidity burden analysis, a cumulative burden index was constructed by summing the number of diagnosed conditions (0 = no disease, 1 = one disease, 2 = two diseases, 3 = three diseases). Poisson regression with robust standard errors was applied to examine factors associated with multimorbidity burden. All statistical tests were two-sided, with significance set at *p* < 0.05.

## Results

### Basic characteristics

#### Demographic characteristics

Female (59.5%)> male (40.5%), the mean age was 56.70 ± 9.87 years. The population was divided into four age groups: 35–49 years (26.2%), 50–59 years (31.2%), 60–69 years (32.1%), and 70 years or older (10.4%). rural residents (70.7%) > urban residents (29.3%), with the Han ethnicity constituting the majority (99.6%). 

#### Social and economic characteristics

The majority of respondents had an education level below high school (77.0%), with the annual household income of 69.8% being less than RMB 50,000, and 91.7% were married. The proportion of individuals with medical insurance reached as high as 97.1%.

#### Behaviors

Smokers accounted for 19.1%, and alcohol consumers accounted for 10.0%.

Among these, the Han ethnicity and medical insurance coverage rates exhibit excessively high proportions, resulting in extreme data sparsity that leads to complete separation, preventing the model from converging. Therefore, they were not incorporated into subsequent analyses (Table 1).

**Table 1:** Participants’ demographic, socioeconomic characteristics, and lifestyle (*n* = 135,352)

Characteristics		Values	*N*(%)	Characteristics		Values	*N*(%)
Gender	Male	1	54,794(40.5)	Medical insurance	Yes	1	131,440(97.1)
	Female	0	80,558(59.5)		No	0	3912(2.9)
Age group	35 ~ 49	1	35,436(26.2)	Annual family income ≥ 50,000 RMB	Yes	1	40,829(30.2)
	50~	2	42,288(31.2)		No	0	94,523(69.8)
	60~	3	43,509(32.1)	Married	Yes	1	124,078(91.7)
	70~	4	14,119(10.4)		No	0	11,274(8.3)
				Smoking	Yes	1	25,849(19.1)
Area	Urban	0	39,629(29.3)		No	0	109,503(80.9)
	Rural	1	95,723(70.7)	Drinking	Yes	1	13,490(10.0)
Nationality	Han	1	134,825(99.6)		No	0	121,862(90.0)
	Others	0	527(0.4)	Prevalence of HTN	-	-	62,521(46.2)
High school or above	Yes	1	31,157(23.0)	Prevalence of T2DM	-	-	24,801(18.3)
	No	0	104,195(77.0)	Prevalence of dyslipidemia	-	-	22,492(16.6)

### Multivariate linear regression analysis of influencing factors on obesity indicators (BMI and WC)

BMI and WC are both commonly used indicators to measure the degree of obesity. The BMI and WC of the participants were 24.07 ± 3.19 kg/m^2^ and 82.52 ± 9.17 cm, respectively.

The study conducted multiple linear regression analyses using BMI and WC as dependent variables, respectively, with the models demonstrating good overall fit. Collinearity diagnostics indicated that all independent variables had variance inflation factors (VIF) < 5 (1.022–1.575), confirming no multicollinearity; the Durbin-Watson values were close to 2 (1.668 for the BMI model and 1.631 for the WC model), and the residuals were independent and normally distributed. Although the BMI model exhibited low explanatory power (adjusted R² = 0.005) while the WC model showed acceptable explanatory power (adjusted R² = 0.053), statistical inference of the regression coefficients remained reliable given the large sample size (*n* > 135,000) and the Central Limit Theorem (Table 2).

**Table 2:** Multivariate linear regression analysis of factors associated with BMI and WC

Independent variable	BMI (kg/m²)		WC (cm)	
	B (95% CI)	ß	B (95% CI)	ß
Male vs. Female	0.340 (0.297, 0.384)	0.052	4.073 (3.952, 4.195)	0.218
Age group (per group)	-0.082 (-0.100, -0.063)	-0.025	0.697 (0.646, 0.749)	0.073
Rural vs. Urban	-0.065 (-0.103, -0.027)	-0.009	-1.170 (-1.277, -1.064)	-0.058
High school education or above vs. below high school level	-0.279 (-0.323, -0.235)	-0.037	-0.697 (-0.820, -0.573)	-0.032
Family annual income > 50,000 vs. ≤ 50,000	0.183 (0.144, 0.222)	0.026	0.524 (0.415, 0.633)	0.026
Married vs. Unmarried	-0.065 (-0.127, -0.003)	-0.006	-0.421 (-0.595, -0.247)	-0.013
Smoking vs. No smoking	-0.531 (-0.583, -0.478)	-0.065	-1.086 (-1.233, -0.940)	-0.047
Drinking vs. No drinking	0.204 (0.145, 0.264)	0.019	0.908 (0.740, 1.076)	0.03

*B* non-standardized regression coefficient, *ß* standardized regression coefficient, *CI* confidence interval, *VIF* 0.022–1.575. Adjusted R² BMI = 0.005, WC = 0.053

The variables demonstrated independent positive correlations with higher BMI and WC as follows: male, annual family income ≥ 50,000 RMB, and drinking.

The variables were significantly correlated with lower BMI and WC as follows: rural, high school or above, married, and smoking.

#### Age exhibits an inverse correlation with the rise in BMI and WC

For each additional age group, BMI decreases significantly (B = -0.082; 95% CI: -0.100, -0.063), whereas WC increases significantly (B = 0.697; 95% CI: 0.646, 0.749). This suggests that, despite an overall decline in BMI with advancing age, the risk of central obesity rises.

#### The “paradox” of smoking

Although smoking is significantly associated with lower BMI and WC, given potential confounding factors (such as physical activity and drinking habits) and the weak magnitude of the effect, it should not be interpreted as indicating a health protective effect of smoking.

### Multivariate linear regression analysis of influencing factors on SBP, DBP, FPG, TC, TG, HDL-C, and LDL-C

The SBP, DBP, FPG, TC, TG, HDL-C, and LDL-C were 133.25 ± 19.31 mmHg, 80.00 ± 11.14 mmHg, 6.09 ± 1.64 mmol/L, 4.88 ± 1.16 mmol/L, 1.64 ± 0.99 mmol/L, 1.46 ± 0.42 mmol/L, and 2.70 ± 0.97 mmol/L, respectively.

Take each indicator as the dependent variable one by one, take related factors as independent variables, and include all of them into the model at one time for multiple linear regression analysis. BMI and WC are independent variables. They have different dimensions, so we will perform Z-score standardization on them, which gives them a mean of 0 and a standard deviation of 1. We will calculate the effect of a 1-SD increase on the outcome variable. This makes it easy to directly compare their relative contribution sizes (Table 3).

**Table 3:** Multivariate linear regression analysis of factors on blood pressure, blood glucose, and blood lipids

Independent variable	SBP (mmHg)		DBP (mmHg)		FPG (mmol/L)		TC (mmol/L)		TG (mmol/L)		HDL-C (mmol/L)		LDL-C (mmol/L)	
	B (95% CI)	ß	B(95%CI)	ß	B(95%CI)	ß	B(95%CI)	ß	B(95%CI)	ß	B(95%CI)	ß	B(95%CI)	ß
Male vs. Female	-0.587 (-0.835, -0.338)	-0.015	2.638 (2.489, 2.786)	0.116	-0.029 (-0.051, -0.007)	-0.009	-0.532 (-0.548, -0.517)	-0.225	-0.059 (-0.072, -0.045)	-0.029	-0.185 (-0.190, -0.180)	-0.219	-0.288 (-0.302, -0.274)	-0.146
Age group (per group)	5.606 (5.503, 5.709)	0.280	0.014 (-0.048, 0.075)^NS^	0.001	0.233 (0.223, 0.242)	0.137	0.134 (0.128, 0.141)	0.112	-0.010 (-0.015, -0.004)	-0.009	0.030 (0.028, 0.032)	0.070	0.100 (0.094, 0.105)	0.099
Rural vs. Urban	4.595 (4.382, 4.809)	0.108	1.767 (1.639, 1.894)	0.072	0.085 (0.066, 0.105)	0.024	0.088 (0.075, 0.102)	0.035	-0.012 (-0.023, 0.000)^NS^	-0.006	0.015 (0.010, 0.020)	0.016	0.072 (0.060, 0.084)	0.034
High school education or above vs. below high school level	-1.453 (-1.698, -1.207)	-0.032	-0.196 (-0.342, -0.050)	-0.007	-0.096 (-0.118, -0.074)	-0.025	-0.009 (-0.025, 0.007)^NS^	-0.003	0.053 (0.040, 0.066)	0.023	-0.026 (-0.031, -0.021)	-0.027	-0.011 (-0.025, 0.003)^NS^	-0.005
Family annual income > 50,000 vs. ≤ 50,000	-1.095 (-1.312, -0.878)	-0.026	-0.247 (-0.376, -0.118)	-0.010	-0.056 (-0.075, -0.036)	-0.016	0.073 (0.059, 0.087)	0.029	-0.037 (-0.049, -0.026)	-0.017	-0.023 (-0.027, -00.018)	-0.025	0.098 (0.086, 0.110)	0.046
Married vs. Unmarried	-0.138 (-0.484, 0.208)^NS^	-0.002	0.201 (-0.005, 0.407)^NS^	0.005	-0.012 (-0.044, 0.019)^NS^	-0.002	-0.035 (-0.057, -0.013)	-0.008	0.016 (-0.003, 0.034)^NS^	0.004	-0.003 (-0.010, 0.005)^NS^	-0.002	-0.034 (-0.053, -0.014)	-0.010
Smoking vs. No smoking	-0.936 (-1.229, -0.644)	-0.019	-0.265 (-0.439, -0.091)	-0.009	-0.025 (-0.051, 0.002)^NS^	-0.006	0.005 (-0.013, 0.024)^NS^	0.002	0.096 (0.080, 0.112)	0.038	-0.065 (-0.071, -0.059)	-0.061	0.040 (0.024, 0.057)	0.016
Drinking vs. No drinking	3.756 (3.422, 4.090)	0.058	2.777 (2.577, 2.976)	0.075	0.136 (0.105, 0.166)	0.025	0.139 (0.118, 0.160)	0.036	0.198 (0.180, 0.216)	0.060	0.160 (0.153, 0.167)	0.115	-0.112 (-0.131, -0.093)	-0.034
ZBMI (per SD)	2.214 (2.059, 2.368)	0.115	1.509 (1.417, 1.601)	0.136	0.034 (0.020, 0.047)	0.020	-0.001 (-0.011, 0.009)^NS^	-0.001	0.090 (0.082, 0.098)	0.091	-0.061 (-0.064, -0.058)	-0.147	0.024 (0.015, 0.032)	0.024
ZWC (per SD)	2.554 (2.395, 2.713)	0.132	1.497 (1.402, 1.592)	0.134	0.214 (0.200, 0.229)	0.130	0.022 (0.011, 0.032)	0.018	0.175 (0.166, 0.184)	0.176	-0.077 (-0.081, -0.074)	-0.185	0.027 (0.018, 0.036)	0.028

SD: BMI ~ 3.5 kg/m², WC ~ 10 cm. adjusted R²:SBP = 0.157,DBP = 0.100,FPG = 0.047,TC = 0.055,TG = 0.070,HDL-C = 0.179,LDL = 0.033

*NS* no significant difference

The results demonstrate that all models are statistically significant, with overall goodness-of-fit and Durbin-Watson values meeting the required criteria; no significant multicollinearity was observed (VIF < 10, Tolerance > 0.1), and residuals follow an independent normal distribution.

Association of obesity indicators with blood pressure: WC is more strongly associated with SBP. The SBP model accounts for 15.7% of the variability (adjusted R² = 0.157). After controlling for confounding factors, every 1 SD increase in BMI (3.5 kg/m²) is associated with a rise in SBP by 2.21 mmHg (95% CI: 2.06, 2.37), while every 1 SD increase in WC (approximately 10 cm) corresponds to a rise in SBP by 2.55 mmHg (95% CI: 2.40, 2.71). The standardized effect of WC (β = 0.132) exceeds that of BMI (β = 0.115), suggesting that central obesity contributes slightly more significantly to SBP elevation. The DBP model accounts for 10% of the variability (adjusted R² = 0.100). For every 1 SD increase in BMI and WC, DBP rises by 1.51 mmHg (95% CI: 1.417, 1.601) and 1.50 mmHg (95% CI: 1.402, 1.592), respectively. Their standardized effects are nearly identical (β_BMI_ = 0.136, β_WC_ = 0.134), indicating that both general obesity and central obesity contribute similarly to DBP.

Associations of obesity indicators with FPG and lipid profiles: WC demonstrates consistently stronger associations. The association of WC on FPG far exceeds that of BMI. After controlling for confounding factors, every 1 SD increase in WC and FPG rises by 0.21 mmol/L (95% CI: 0.200, 0.229), with a standardized effect (β = 0.130) approximately 6.5 times greater than that of BMI (β = 0.020). The effect of WC on TG is 1.93 times that of BMI (β_BMI_ = 0.091, β_WC_ = 0.176 ). Both WC and BMI are negatively correlated with HDL-C levels, but WC is more pronounced.(β_BMI_ = -0.147,β_WC_ =-0.185)。.

WC showed a weak positive correlation with TC (B = 0.022 mmol/L; 95% CI: 0.011, 0.032). But there was no significant independent association between BMI and TC. In the LDL model, the associations of both BMI and WC were weak and similar (β_BMI =_ 0.024, β_WC_ = 0.028), and the models exhibited the lowest explanatory power (3.3%), indicating that LDL is less strongly associated with obesity and primarily related to genetic factors and dietary factors.

The association patterns of other covariates are as follows: advanced age, rural residence, and drinking are typically associated with elevated levels of SBP, DBP, FBG, and TC. Higher education and higher income are generally linked to reduced values of SBP, DBP, and FBG. Additionally, smoking exhibits a positive correlation with both higher education attainment and elevated TG levels, which contradicts conventional assumptions and may be attributed to lifestyle interactions specific to certain populations.

### Prevalence of HTN, T2DM, dyslipidemia, and multimorbidity burden, and modified Poisson regression

The study revealed a significant burden of NCDs among the surveyed population, with HTN affecting 46.2% (62,521), T2DM affecting 18.3% (24,801), and dyslipidemia affecting 16.6% (22,492) of the 135,352 participants (Table 1). Since the prevalence of these three diseases exceeds 10%, Modified Poisson regression (GENLIN) is required to prevent the OR from overestimating the true RR (Table 4).

**Table 4:** Modified Poisson regression: adjusted risk ratios (aRR) for hypertension, T2DM, dyslipidemia, and multimorbidity

Independent variable	HTN		T2DM		dyslipidemia		Multimorbidity Burden	
	B	aRR (95% CI)	B	aRR (95% CI)	B	aRR (95% CI)	B	aRR (95% CI)
Age group								
group 4 vs. 1	2.838	17.079 (16.447,17.734)	1.418^#^	4.127^#^	0.588	1.800 (1.626, 1.993)	1.544	4.682 (4.524, 4.844)
group 3 vs. 1	2.619	13.727 (13.253,14.218)	1.350^#^	3.856^#^	0.629	1.875 (1.724, 2.039)	1.341	3.824 (3.707, 3.945)
group 2 vs. 1	2.221	9.221 (8.892, 9.563)	1.024^#^	2.784^#^	0.414	1.513 (1.393, 1.644)	0.895	2.447 (2.369, 2.527)
Male vs. Female	0.056	1.058 (1.036, 1.079)	0.066	1.069 (1.029, 1.110)	-0.250	0.779 (0.732, 0.829)	0.023	1.023 (1.003, 1.043)
Rural vs. Urban	-0.134	0.875 (0.860, 0.890)	-0.264	0.768 (0.743, 0.794)	-0.498	0.608 (0.577, 0.641)	-0.267	0.766 (0.753, 0.779)
High school education or above vs. below high school level	0.001	1.00 (0.979, 1.023)^NS^	0.016	1.016(0.976, 1.059)^NS^	0.665	1.945 (1.838, 2.059)	0.121	1.128 (1.105, 1.152)
Family annual income > 50,000 vs. ≤ 50,000	-0.003	0.997(0.978, 1.016)^NS^	0.049	1.051(1.014, 1.088)	0.464	1.590 (1.509, 1.675)	0.088	1.092 (1.073, 1.112)
Married vs. Unmarried	0.009	1.009(0.982, 1.037)^NS^	0.016	1.016 (0.964, 1.071)^NS^	-0.334	0.716^#^	-0.014	0.987(0.961, 1.013)^NS^
Smoking vs. No smoking	-0.032	0.969(0.945, 0.994)	-0.049	0.953 (0.907, 1.000)^NS^	-0.137	0.872 (0.804, 0.946)	-0.058	0.944 (0.921, 0.967)
Drinking vs. No drinking	0.119	1.126(1.095, 1.158)	-0.161	0.851 (0.803, 0.902)	0.101	1.106 (1.014, 1.206)	0.056	1.058 (1.030, 1.087)
Obesity types								
group 4 vs. 1	0.655	1.926 (1.885, 1.967)	0.454	1.574 (1.513, 1.638)	0.338	1.402 (1.320, 1.489)	0.679	1.973 (1.934, 2.012)
group 3 vs. 1	0.361	1.434 (1.381, 1.490)	0.133	1.143 (1.061, 1.231)	0.265	1.304 (1.175, 1.446)	0.343	1.409 (1.359, 1.461)
group 2 vs. 1	0.306	1.358 (1.319, 1.399)	0.402	1.495 (1.420, 1.574)	0.097	1.102 (1.014, 1.198)	0.359	1.432 (1.393, 1.471)
multimorbidity 1	0.495 (T2DM)	1.640 (1.610, 1.671)	0.831 (HTN)	2.296 (2.218, 2.377)	0.748 (HTN)	2.113 (1.999, 2.234)	-	-
multimorbidity 2	0.390 (dyslipidemia)	1.477 (1.439, 1.516)	0.571 (dyslipidemia)	1.770 (1.687, 1.856)	0.673 (T2DM)	1.961 (1.847, 2.081)	-	-

NS, The 95% confidence interval (CI) of aRR includes 1, with no statistical significance

Age group: group 1 35–49 years, group 2 50–59 years, group 3 60–69 years, group 4 > 70 years

Obesity types: group1=“both WC and BMI normal”, group2=“isolated WC elevation”, group3=“isolated BMI elevation”, group4=“both WC and BMI elevation”

#: This variable exhibits quasi-complete separation (spillover), with large standard errors and unstable parameter estimates; it is provided for reference only

Using HTN, T2DM, and dyslipidemia as dependent variables, the model incorporated an obesity type variable in addition to general factors to analyze the correlations between different obesity types and these three diseases. The variable was categorized into four groups based on BMI and WC: Group 1, both BMI and WC normal (38.3%); Group 2: isolated WC elevation (13.3%); Group 3: elevated BMI with normal WC (7.7%) ; Group 4: elevated BMI and WC (40.4%). Correlation analysis demonstrated significant positive correlations between HTN, T2DM, and dyslipidemia in all pairwise combinations (*p* < 0.001), indicating a pronounced multimorbidity clustering phenomenon. Therefore, the analysis incorporated the other two conditions for multimorbidity adjustment to obtain more robust independent effect estimates.

#### Modified Poisson regression of HTN T2DM and dyslipidemia

The HTN model indicated that all obesity types were independently associated with elevated HTN risk, with varying associations depending on the obesity subtype: mixed obesity (aRR = 1.926), elevated BMI alone (aRR = 1.434), and elevated WC alone (aRR = 1.358).

Regarding other factors, age (aRR = 17.079) was the strongest factor independently associated with HTN, with the strength of the association increasing exponentially with age > 70 years group. Male and drinking were also associated with an elevated risk of HTN, whereas rural residence and smoking were associated with a lower risk. No significant associations were observed for education or income after adjusting for comorbidities.

In the T2DM model, age was not reported due to complete separation. The results indicated that all obesity types were independently associated with an increased risk of T2DM. Mixed obesity (aRR = 1.574, 95% CI: 1.513, 1.638), elevated WC alone (aRR = 1.495, 95% CI: 1.420, 1.574), and elevated BMI alone (aRR = 1.143, 95% CI: 1.061, 1.231) demonstrated a significant association with T2DM.

Regarding other factors, males (aRR = 1.069, 95% CI: 1.029, 1.110), urban residents (aRR = 1.302, 95% CI: 1.260, 1.346), and higher income (aRR = 1.051, 95% CI: 1.014, 1.088) were independently associated with an increased risk of T2DM, whereas drinking (aRR = 0.851, 95% CI: 0.803, 0.902) was associated with a lower risk.

The dyslipidemia model indicated that: mixed obesity (aRR = 1.402, 95% CI: 1.320–1.489), elevated BMI alone (aRR = 1.304, 95% CI: 1.175–1.446), and elevated WC alone (aRR = 1.102, 95% CI: 1.014–1.198) demonstrated a notable association with an increased risk of dyslipidemia.

Other factors: The risk of dyslipidemia significantly increased with age, peaking in the 60–69 years group (aRR = 1.875, 95% CI: 1.724, 2.039) and showing a slight decrease in the > 70 years group (aRR = 1.800, 95% CI: 1.626, 1.993). Higher education level (aRR = 1.945, 95% CI: 1.838, 2.059), high income (aRR = 1.590, 95% CI: 1.509, 1.675), and drinking (aRR = 1.106, 95% CI: 1.014, 1.206) were independently associated with an increased risk of dyslipidemia, whereas male sex (aRR = 0.779, 95% CI: 0.732, 0.829), rural residence (aRR = 0.608, 95% CI: 0.577, 0.641), and smoking (aRR = 0.872, 95% CI: 0.804, 0.946) were associated with a lower risk.

#### Modified Poisson regression of multimorbidity burden

A multimorbidity burden index was established based on the multimorbidity status of the three diseases, with scores assigned as follows: 0 (no disease) (49.4%), 1 (one disease) (41.7%), 2 (two comorbidities) (8.0%), and 3 (three comorbidities) (0.9%).

Examination of the variable distribution revealed a variance-to-mean ratio of 1.02, indicating slight underdispersion. Consequently, Poisson regression with robust standard errors was employed to analyze factors associated with the multimorbidity burden (Table 4). The results are as follows:

Elevated WC alone (aRR = 1.432, 95% CI: 1.393, 1.471), elevated BMI alone (aRR = 1.409, 95% CI: 1.359, 1.461), and mixed obesity (aRR = 1.973, 95% CI: 1.934, 2.012) were independently associated with an increased multimorbidity burden. Mixed obesity was associated with an almost twofold increase in multimorbidity burden.

Regarding sociodemographic and behavioral factors, age was the strongest factor associated with the multimorbidity burden. The association strength was highest in the > 70 years group (aRR = 4.682, 95% CI: 4.524, 4.844). Higher education (aRR = 1.128, 95% CI: 1.105, 1.152), higher income (aRR = 1.092, 95% CI: 1.073, 1.112), and male (aRR = 1.023, 95% CI: 1.003, 1.043) were weakly positively associated with multimorbidity burden, whereas rural residence (aRR = 0.766, 95% CI: 0.753, 0.779) was associated with a lower burden. Additionally, drinking was associated with an increased burden (aRR = 1.058, 95% CI: 1.030, 1.087), while smoking was associated with a decreased burden (aRR = 0.944, 95% CI: 0.921, 0.967), which may be related to lower body weight among smokers.

## Discussion

This large community-based study systematically compared the associations of BMI and WC with HTN, T2DM, and dyslipidemia. The principal findings can be summarized as follows: (1) both BMI and WC were positively and independently associated with the risk of all three NCDs, but the strength and pattern of these associations differed by disease type. (2) WC demonstrated a numerically larger association with SBP, FBG, TG, and HDL-C in standardized analyses, while the two indicators showed comparable associations with DBP and LDL-C. (3) The regression model analysis revealed that three obesity subtypes—mixed obesity, elevated BMI alone, and elevated WC alone (central obesity)—all independently associated with an increased risk of HTN, T2DM, and dyslipidemia. (4) Combined elevation of BMI and WC (mixed obesity) was associated with a significant risk across all outcomes, with nearly a two-fold increase in multimorbidity burden (aRR = 1.973). (5) Age was the strongest independent correlate of all three diseases and their multimorbidity. (6) Smoking exhibits an “anomalously protective” association. Higher education levels, higher income, and male sex are associated with an increased risk of certain metabolic diseases, whereas rural residents demonstrate a protective association. These findings extend the existing literature by providing head-to-head comparisons of BMI and WC using both categorical and standardized continuous approaches in a single large Asian population.

### Differential associations of obesity phenotypes with metabolic diseases

A key finding of this study is that the associations of BMI and WC with NCDs exhibit distinct patterns depending on the specific disease outcome. For HTN and dyslipidemia, isolated BMI elevation was significantly associated with an increased risk. In contrast, for T2DM, isolated WC elevation demonstrated a significant association. This differential pattern is biologically plausible and consistent with the distinct pathophysiological pathways linking general obesity and central obesity to metabolic outcomes [9].

Visceral white adipose tissue (vWAT), which is specifically captured by WC, exhibits greater metabolic activity than subcutaneous adipose tissue. vWAT is characterized by higher rates of lipolysis and greater secretion of pro-inflammatory cytokines (e.g., IL-6, TNF-alpha), and increased production of adipokines such as resistin and leptin, all of which directly promote IR [8]. The portal theory further posits that free fatty acids released from visceral adipocytes enter the portal circulation directly, exposing the liver to high concentrations of free fatty acids, thereby impairing hepatic insulin signaling, increasing hepatic gluconeogenesis, and promoting triglyceride synthesis [18].

#### These mechanisms explain why WC, as a proxy for visceral adiposity, demonstrates a significant association with T2DM risk in our study

 In contrast, BMI primarily reflects overall adiposity and is more closely linked to hemodynamic mechanisms of blood pressure elevation. Excess total body fat increases blood volume, cardiac output, and activates the renin-angiotensin-aldosterone system (RAAS), leading to elevated blood pressure [11]. Additionally, obesity-related leptin resistance (LR) may contribute to sympathetic nervous system activation and further increase arterial pressure [19]. These hemodynamic effects provide a biological rationale for the significant association observed between isolated BMI elevation and HTN in our study.

Although HTN, T2DM, and dyslipidemia are intercorrelated components of cardiometabolic risk, analyzing them separately offers three advantages: First, it allows for the detection of differential associations between BMI and WC and specific pathophysiological domains; Second, it provides actionable evidence for targeted screening in resource-limited primary care settings; Third, it avoids the information loss inherent in composite outcomes such as metabolic syndrome, which may obscure the unique contribution of central adiposity to individual disease endpoints.

### Standardized comparison of WC and BMI: insights from multivariate linear regression

The use of Z-score standardization in our linear regression models allowed direct comparison of the effect sizes of BMI and WC on continuous metabolic parameters. First, WC showed a stronger standardized association with SBP than BMI, while the two indicators were nearly identical for DBP. This differential effect on SBP versus DBP has important clinical implications. SBP is predominantly determined by large artery stiffness and pulse wave velocity, which are more directly influenced by visceral adiposity-related inflammation and endothelial dysfunction [11]. DBP, on the other hand, is more closely tied to peripheral vascular resistance (PVR) [6], which is influenced by both general and central obesity through overlapping mechanisms [20, 21].

Second, the standardized effect of WC on FPG was approximately 6.5 times that of BMI. It underscores the primacy of central adiposity in glucose metabolism. This finding aligns with the meta-analysis by Jayedi et al. [9]. Similarly, WC showed a substantially stronger association with TG and a more pronounced inverse association with HDL-C. Hypertriglyceridemia and low HDL-C are a hallmark of IR and the metabolic syndrome [22]. These results suggest that central obesity exerts a greater influence on the dyslipidemia phenotype.

Third, both BMI and WC showed weak and similar associations with LDL-C, and the overall model explained only 3.3% of LDL-C variability. This finding is consistent with the understanding that LDL-C levels are primarily determined by genetic factors and dietary cholesterol intake rather than obesity per se [23].

### The synergistic risk of mixed obesity

Our analysis of BMI-WC combined categories revealed that individuals with both elevated BMI and elevated WC (mixed obesity) had the highest risk for all three NCDs and a near doubling of the multimorbidity burden. Importantly, individuals with a normal BMI but elevated WC (normal-weight central obesity) still exhibited significantly elevated risks for HTN and T2DM, compared with those with both a normal BMI and WC.

The synergistic effect of mixed obesity likely reflects the convergence of hemodynamic and metabolic pathways: elevated BMI increases blood volume and cardiac workload [24], while elevated WC adds the metabolic burden of visceral adiposity, including IR, inflammation, and dyslipidemia. The combination of these two obesity phenotypes creates a substantially elevated multimorbidity burden.

These findings underscore the importance of measuring both BMI and WC in clinical practice, as reliance on BMI alone would miss a substantial proportion of individuals at elevated risk due to central obesity.

### Sociodemographic and behavioral correlates

Our study identified several sociodemographic and behavioral factors independently associated with BMI, WC, and NCD risk. Male, urban residence, higher income, and drinking were associated with higher WC, while rural residence, higher education, and marriage were associated with lower WC. These findings are consistent with previous studies in Chinese populations [4] and reflect the complex interplay between socioeconomic development, lifestyle changes, and obesity patterns in transitional economies.

Age demonstrated an intriguing pattern: while BMI decreased with advancing age, WC increased. This age-related redistribution of body fat from general to central depots is well-documented [25] and may reflect hormonal changes (e.g., declining growth hormone and sex hormones), reduced physical activity, and increased visceral fat accumulation with aging. The clinical implication is that elderly individuals may have a normal BMI but nevertheless harbor significant central obesity and its attendant metabolic risks.

### The smoking paradox

Our study found that smoking was associated with lower BMI and WC and appeared to be “protective” in some disease models (e.g., dyslipidemia aRR = 0.872, multimorbidity aRR = 0.944). This finding, often termed the “smoking paradox” or “obesity paradox,” requires careful interpretation and should not be misconstrued as evidence of any health benefit of smoking.

Several mechanisms may explain this paradoxical observation. First, apart from its anorectic action, nicotine also modulates energy expenditure by regulating brown adipose tissue (BAT) thermogenesis and white adipose tissue (WAT) browning [26]. Our linear regression results confirm that smoking is independently associated with lower BMI (B = -0.531) and WC (B = -1.087).

Second, a healthy survivor effect may be operating: individuals who continue to smoke despite having metabolic diseases may represent a healthier subset, while those who quit smoking due to illness are classified as non-smokers, creating a spurious protective association.

Third, residual confounding by unmeasured factors such as physical activity, dietary habits, and socioeconomic status cannot be excluded.

It is critical to emphasize that the apparent protective association of smoking is a statistical artifact. Smoking’s harmful effects on cardiovascular health include endothelial damage, oxidative stress, pro-inflammatory effects, and direct carcinogenicity. These harms far outweigh any modest effect on body weight. Public health messages must remain unequivocal: smoking cessation should be promoted for all individuals, regardless of body weight status, and smoking should never be encouraged as a weight management strategy.

### Public health and clinical implications

Our findings have several important implications for clinical practice and public health policy. First, the measurement of WC should be integrated into routine clinical screening alongside BMI. Our data demonstrate that WC provides incremental value in risk stratification, especially for identifying individuals at elevated risk for T2DM and those with the metabolically obese normal-weight phenotype.

Second, targeted interventions should consider the differential risk profiles associated with BMI and WC. For populations at high risk of T2DM, WC reduction should be a primary therapeutic target. For HTN and dyslipidemia management, both overall weight loss and WC reduction should be pursued simultaneously. Lifestyle interventions incorporating dietary modification, physical activity, and behavioral support have been shown to effectively reduce both BMI and WC [27, 28].

Third, our identification of high-risk subgroups—men, urban residents, high-income individuals, smokers, and drinkers—provides actionable targets for public health interventions. These groups should be prioritized for screening and lifestyle modification programs. Given the high prevalence of HTN (46.2%), T2DM (18.3%), and dyslipidemia (16.6%) in our study population, population-wide strategies, including health education, regular screening, and environmental interventions to promote healthy lifestyles, are urgently needed.

Fourth, the high multimorbidity burden associated with mixed obesity underscores the need for integrated management of cardiometabolic risk factors. Rather than addressing each disease in isolation, healthcare systems should adopt a holistic approach that simultaneously targets obesity, HTN, diabetes, and dyslipidemia through coordinated interventions.

China is undergoing rapid urbanization, nutritional transition, and a shift toward sedentary lifestyles, which have contributed to an increasing prevalence of central obesity even among individuals with normal BMI. In this context, the prominent association of WC with T2DM and glucose abnormalities—and the notable presence of normal-weight central obesity in our sample—reflects these broader epidemiological trends. Thus, the distinct association pattern of WC for NCDs observed in our study is not merely a statistical finding but may have growing clinical and public health relevance as the population continues to transition.

### Strengths and limitations

This study has several notable strengths. First, the large sample size (*n* = 135,352) with rigorous multistage stratified cluster random sampling ensures high statistical power and generalizability to the population of Southeast China. Second, the comprehensive assessment of both continuous and categorical obesity indicators, combined with standardized effect size comparisons, provides a robust analytical framework. Third, the simultaneous examination of three major NCDs and their multimorbidity offers a holistic view of the obesity-NCD relationship. Fourth, the use of modified Poisson regression for prevalence outcomes (> 10%) avoids the overestimation bias inherent in logistic regression.

However, several limitations should be acknowledged. First, the cross-sectional design precludes causal inference; longitudinal studies are needed to establish temporal relationships between obesity indicators and NCD incidence. Second, fasting blood samples were collected via fingertip puncture rather than venous sampling, which may have introduced minor measurement errors in glucose and lipid profiles. Misclassification of diabetes type is possible but unlikely to affect the main associations given the age range and the very low prevalence of non-T2DM in this population. Third, data on dietary intake, physical activity, and genetic factors were not collected, potentially leaving residual confounding. Fourth, the study population was predominantly Han (99.6%) and rural (70.7%), which may limit generalizability to more ethnically diverse or urbanized populations. Fifth, the nearly universal medical insurance coverage (97.1%) in our sample differs from many other regions, potentially affecting the generalizability of findings related to healthcare access. Sixth, the present study focused on overall associations in the whole sample. Subgroup analyses by sex, age, urban/rural residence, or lifestyle factors were not performed due to space constraints and the risk of multiple comparisons; these will be addressed in future separate studies, and restricted cubic splines will be used to explore non-linear relationships. Seventh, we did not perform direct statistical comparisons (e.g., interaction tests, or nested model comparisons) to determine whether the risk magnitude of BMI was statistically significantly different from that of WC. Therefore, the observed differential associations should be interpreted as distinct patterns rather than results of a direct head-to-head statistical test. Eighth, the complex multistage stratified cluster sampling design may have introduced clustering effects. We did not apply sampling weights or adjust for design effects in the regression models because the primary goal was to compare exposure–outcome associations, not to produce population-representative prevalence estimates. However, future studies should consider survey-weighted analyses to confirm generalizability.

Despite these limitations, the consistency of our findings with previous research and the biological plausibility of the observed associations lend confidence to our conclusions. Future prospective studies with comprehensive covariate assessment, including dietary, physical activity, and genetic data, are warranted to confirm and extend these findings.

## Conclusion

This study of 135,352 Southeast Chinese residents demonstrates that both WC and BMI are significantly associated with HTN, T2DM, and dyslipidemia, with WC showing a prominent association with T2DM. Mixed obesity confers the highest risk and nearly doubles the multimorbidity burden.

The prevalence rates of NCDs were high in this population, underscoring the urgent need for effective obesity prevention and control strategies. Both WC and BMI varied significantly by sex, age, region, education level, and lifestyle factors, with higher WC specifically associated with male, urban residence, smoking, and drinking.

These findings have important implications for clinical practice and public health policy. The integration of WC measurement into routine screening alongside BMI is recommended. This can help avoid missing a substantial proportion of individuals at elevated risk due to central obesity. Targeted interventions should prioritize high-risk subgroups, including men, urban residents, and individuals with mixed obesity. Future prospective studies are warranted to establish causal relationships and to evaluate the effectiveness of WC-centered screening and intervention programs in reducing the burden of NCDs.

## Supplementary Information

Supplementary Material 1.

## Data Availability

No datasets were generated or analysed during the current study.

## Abbreviations

WC: Waist circumference
BMI: Body mass index
NCDs: Noncommunicable diseases
IR: Insulin resistance
HTN: Hypertension
T2DM: Type 2 diabetes mellitus
SBP: Systolic blood pressure
DBP: Diastolic blood pressure
FPG: Fasting plasma glucose
TC: Total cholesterol
TG: Triglyceride
LDL-C: Low-density lipoprotein cholesterol
HDL-C: High-density lipoprotein cholesterol
SD: Standard deviation
