# Supplementary material for: Waist circumference shows a significant association with the risk of noncommunicable diseases: a cross-sectional study from a large community sample in Southeast China
Source: BMC Public Health. 2026 Jul 9;26:2871. doi: 10.1186/s12889-026-28417-7 (PMC13647853; doi:10.1186/s12889-026-28417-7)
Supplement: Supplementary file 1 [file 12889_2026_28417_MOESM1_ESM.docx]

# **Supplementary Material: Consistency Assessment of Outcome Definitions and Sensitivity Analysis**

## **1. Consistency Assessment of Outcome Definitions**

Since this study simultaneously used objective measurements and self-reported hospital diagnoses to define hypertension, T2DM, and dyslipidemia, we evaluated the consistency between the two information sources and their potential impact on the results. Specifically, we calculated the Kappa coefficient for each disease and reported the proportional distribution of the three diagnostic sources. The detailed steps were as follows: 1. Generate outcome definition variables; 2. Calculate the prevalence (frequency and percentage) of each definition; 3. Cross-tabulation: examine the consistency between self-reported and objective measurements. The results are presented in the table below.

**Table 1. Distribution of diagnostic sources for three diseases**

| **Disease** | **Objective measurement only** | **Self-report only** | **Both objective and self-report positive** | **Objective or self-report positive (original definition)** | **Kappa (95% CI)** |
| --- | --- | --- | --- | --- | --- |
| Hypertension | 19.2% | 9.0% | 17.6% | 46.2% | 0.355 (0.349–0.361) |
| T2DM | 8.4% | 3.0% | 6.8% | 18.2% | 0.481 (0.473–0.489) |
| Dyslipidemia | 33.9% | 1.9% | 2.5% | 38.3% | 0.046 (0.042–0.050) |

Note: Kappa < 0.20 indicates very low agreement, 0.21–0.40 fair agreement, 0.41–0.60 moderate agreement.

The Kappa coefficient for hypertension was 0.355, indicating fair agreement between objective measurements and self-reports; for T2DM, the Kappa was 0.481, also indicating moderate agreement; for dyslipidemia, the Kappa was only 0.046, indicating very low agreement, primarily due to the extremely low self-report positive rate (only 4.4%), of which self-report-only cases accounted for merely 1.9%. Nevertheless, since the original definition of dyslipidemia (Model A) relied mainly on objective measurements (36.4%), the contribution of self-reports was minimal, and thus the impact on the primary analysis was limited. Based on the above distribution, we further conducted sensitivity analyses (see Section 3.6) to verify the robustness of the results under different diagnostic source definitions.

## **2. Sensitivity Analysis: Associations between Obesity Phenotypes and NCDs under Different Outcome Definitions**

To evaluate potential misclassification bias arising from the combined use of objective measurements and self-reported diagnoses, we performed sensitivity analyses for hypertension, T2DM, and dyslipidemia using three outcome definitions:

- **Model A (original definition)**: Objective measurement **or** self-reported diagnosis positive
- **Model B**: Self-reported diagnosis positive only
- **Model C**: Objective measurement and self-reported diagnosis both positive

Due to the low self-report positive rates (only 1.9% for dyslipidemia), the estimation precision of Models B and C decreased; however, the relative risk order of obesity phenotypes remained consistent across all models. Overall, outcome misclassification did not materially bias the main conclusions. Detailed results are presented in Table 2.

**Table 2. Adjusted risk ratios (aRR, 95% CI) for obesity phenotypes and hypertension, T2DM, and dyslipidemia under different outcome definitions**

| **Disease** | **Obesity phenotype** | **Model A**  **(Objective or self-report)** | **Model B**  **(Self-report only)** | **Model C**  **(Both positive)** |
| --- | --- | --- | --- | --- |
| Hypertension | both WC and BMI elevation | 1.694(1.671–1.717) | 2.039 (1.996–2.083) | 2.182 (2.121–2.245) |
|  | isolated BMI elevation | 1.335(1.303–1.368) | 1.464 (1.409–1.521) | 1.521 (1.446–1.599) |
|  | isolated WC elevation | 1.348(1.322–1.373) | 1.405 (1.363–1.447) | 1.429 (1.373–1.488) |
| T2DM | both WC and BMI elevation | 1.840(1.791–1.891) | 1.935 (1.861–2.013) | 2.074 (1.975–2.177) |
|  | isolated BMI elevation | 1.250(1.188–1.315) | 1.260 (1.169–1.359) | 1.240 (1.128–1.362) |
|  | isolated WC elevation | 1.472 (1.419–1.527) | 1.620 (1.538–1.706) | 1.731 (1.623–1.845) |
| Dyslipidemia | both WC and BMI elevation | 1.722 (1.693–1.751) | 1.768 (1.667–1.876) | * |
|  | isolated BMI elevation | 1.331 (1.293–1.370) | 1.394 (1.255–1.547) | * |
|  | isolated WC elevation | 1.420 (1.387–1.453) | 1.269 (1.167–1.379) | * |

*Note: For dyslipidemia, the number of cases positive for both (Model C) accounted for only 2.5% (n = 3,229), resulting in unstable model estimates; therefore, specific values are not reported. However, the direction of point estimates was consistent with Model A.
